# Effects of Nano Zinc Oxide Supplementation on Oxidative Stress and Selected Metabolic Parameters During the Transition Period in Lacaune Ewes

**DOI:** 10.1002/vms3.71149

**Published:** 2026-08-07

**Authors:** Gaye Bulut, Neşe Hayat Aksoy, Caner Ozturk

**Affiliations:** ^1^ Faculty of Veterinary Medicine Department of Obstetrics and Gynecology Aksaray University Aksaray Türkiye; ^2^ Faculty of Veterinary Medicine Department of Veterinary Biochemistry Aksaray University Aksaray Türkiye; ^3^ Faculty of Veterinary Medicine Department of Reproduction and Artificial insemination Aksaray University Aksaray Türkiye

**Keywords:** antioxidant status, Lacaune ewes, nano zinc oxide, oxidative stress, transition period

## Abstract

**Background:**

The transition period in small ruminants is characterized by marked metabolic and oxidative changes. Nano zinc oxide (nZnO) has potential antioxidant‐related properties, but its effects in dairy‐type ewes remain unclear.

**Objectives:**

This study evaluated the effects of nZnO supplementation on oxidative stress and selected metabolic parameters in pregnant Lacaune ewes during the transition period.

**Methods:**

Fourteen clinically healthy pregnant ewes were randomly assigned to a control group or an nZnO group (25 mg/kg body weight). Blood samples were collected 21 days before lambing, within 1–12 h after lambing and 21 days postpartum. Serum total antioxidant status (TAS), total oxidant status (TOS), oxidative stress index (OSI) and selected biochemical variables were measured. Data were analysed using a linear mixed model for repeated measures.

**Results:**

No significant effects of treatment, time or treatment × time interaction were detected for TAS, TOS or OSI (*p* > 0.05), although non‐significant numerical differences were observed. Significant effects of time were observed for albumin (*p* = 0.006), ALT, AST, BUN, total calcium, total protein and triglycerides (all *p* < 0.001). Treatment effects were detected for BHBA (*p* = 0.009), phosphorus (*p* = 0.035) and total cholesterol (*p* = 0.035), and total cholesterol also showed a treatment × time interaction (*p* = 0.033). No significant effects were detected for NEFA.

**Conclusions:**

At the tested dose, nZnO did not significantly alter oxidative stress indices. The effects on selected metabolic variables should be interpreted cautiously because of the small sample size and lack of zinc status assessment. Larger multi‐dose studies are needed before practical recommendations can be made.

## Graphical abstract

Nano zinc oxide supplementation did not significantly affect TOS, TAS or OSI in transition period Lacaune ewes. However, treatment related responses were observed for BHBA, phosphorus and total cholesterol, suggesting limited metabolic effects that require cautious interpretation.

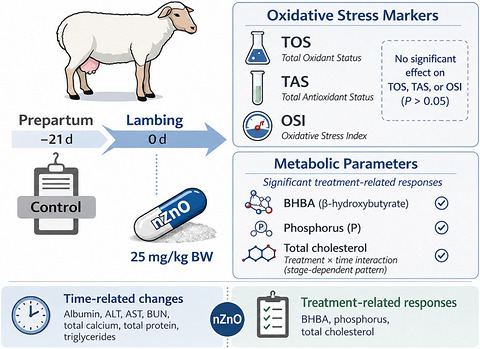

## 1 Introduction

Zinc (Zn) is the second most abundant trace element in the body after iron and is involved in a wide range of biochemical and physiological processes. It plays an important role in immune function, reproduction, growth and the regulation of oxidative stress (Mir et al. 2020; Yatoo et al. 2013). As a cofactor for more than 300 enzymes involved in DNA and protein synthesis, cell division and tissue repair, zinc is essential for normal development and health. Zinc deficiency has been associated with growth retardation, immune dysfunction, anorexia, skin lesions and impaired reproductive performance in both humans and animals (Kumar et al. 2011; Prasad 2012).

In animal nutrition, the form and bioavailability of dietary zinc can directly influence health and performance. Recent advances in nanotechnology have led to the use of nano zinc oxide (nZnO), which has a smaller particle size and a larger surface area than conventional inorganic zinc and may therefore be absorbed more efficiently and interact more effectively with cellular metabolic pathways (Abdelnour et al. 2021; Swain et al. 2016). Experimental studies suggest that nano zinc preparations are more bioavailable than conventional forms and may reach target tissues more readily. Several reports have also indicated that these preparations exert antioxidant effects (Abdollahi et al. 2020; Alijani et al. 2020; Li et al. 2025). However, the biological response to nZnO is not uniform across studies and should not be interpreted as universally beneficial (Rahman et al. 2022,; Yang et al. 2025; Zarghami et al. 2025). Recent literature indicates that the effects of ZnO nanoparticles may vary according to particle characteristics, dose, duration of exposure, animal species, physiological stage, feeding conditions and basal mineral status (Rahman et al. 2022; Yang et al. 2025). Although ZnO nanoparticles may exert antioxidant, antimicrobial, immunomodulatory and growth‐related effects, their use should be balanced against possible toxicological concerns and the need for dose optimization (Rahman et al. 2022; Yang et al. 2025). Recent ruminant data also show that some responses to nZnO supplementation may remain numerical rather than statistically significant, further supporting cautious interpretation in transition‐period studies (Zarghami et al. 2025). In ruminants, supplementation with zinc oxide nanoparticles has been associated with improvements in performance, immunity, antioxidant status and liver‐related enzyme activity (Li et al. 2025; Mansour et al. 2023). In lambs, zinc oxide nanoparticles have been reported to increase antioxidant capacity and reduce oxidative stress index (OSI), accompanied by changes in alanine aminotransferase and aspartate aminotransferase activities. In Ossimi ewes, zinc nanoparticles, alone or in combination with selenium, improved productive performance and several biochemical indices during late gestation and early lactation (Abd El Rahim et al. 2023; Hosseini‐Vardanjani et al. 2020; Ibrahim et al. 2025).

Zinc oxide nanoparticles may also influence rumen microbiota, ruminal function and tissue structure, and they have shown inhibitory effects against several pathogenic microorganisms, including *Escherichia coli* (Ahmed et al. 2024; Dunière et al. 2021; Petrič et al. 2024; Yang et al. 2025). However, their potential toxic effects should also be considered. High doses of zinc oxide nanoparticles may alter mitochondrial function, increase intracellular calcium accumulation, promote reactive oxygen species generation in the liver and increase serum transaminase activities. Experimental studies have also suggested dose‐related damage in the liver and kidneys, as well as stage dependent hepatotoxicity (Najafzadeh et al. 2013; Tang et al. 2016). These findings indicate that nZnO supplementation cannot be assumed to be universally beneficial and that its effects should be evaluated under specific physiological, nutritional and exposure‐related conditions (Fatima et al. 2024).

From the perspective of reproductive physiology, the transition period is a critical stage characterized by profound metabolic and physiological adaptations, during which homeostatic balance becomes particularly vulnerable (Cabiddu et al. 2020; Drackley 1999). During this period, oxidative stress indices and metabolic indicators such as total oxidant status (TOS), total antioxidant status (TAS), non‐esterified fatty acids (NEFA) and beta hydroxybutyrate are commonly evaluated (Meli et al. 2025). Zinc supplementation during the transition period has been reported to support antioxidant capacity and may help reduce postpartum complications (Chen et al. 2023). Nevertheless, the transition period is also associated with considerable physiological and metabolic variation influenced by factors such as parity and litter size, which may make subtle nutritional effects difficult to detect, particularly in small experimental groups (Chagas et al. 2023; Zhang and Hartmann 2023).

Despite the growing interest in nano zinc supplementation, data in dairy‐type sheep breeds remain limited and supplementation strategies vary across studies. Information is especially limited for Lacaune ewes during the transition period, and previous studies have not sufficiently clarified whether nZnO can meaningfully modulate oxidative stress indices while also affecting energy, protein, lipid, mineral and liver‐related metabolic variables in this breed. Therefore, the present study aimed to evaluate the effects of nZnO supplementation in pregnant Lacaune ewes during the transition period on oxidative stress indices and selected metabolic parameters. In particular, TOS, TAS and OSI were assessed together with selected indicators of energy, protein, lipid, mineral and liver‐related metabolism. We hypothesized that nZnO supplementation would influence oxidative stress indices and selected metabolic variables during the transition period, but that these responses may vary according to physiological stage.

## 2 Materials and Methods

### 2.1 Experimental Animals and Study Design

A total of 14 clinically healthy pregnant nulliparous Lacaune ewes were included in the study and randomly allocated to two groups: a control group (*n* = 7) and an nZnO‐supplemented group (*n* = 7). Animals were selected according to the presence of pregnancy, nulliparous status and a body condition score between 3.0 and 3.5 at enrolment. Pregnancy had been confirmed during routine farm examination at approximately 4.0–4.5 months of gestation. Because only the presence of pregnancy was recorded at that stage, litter size was not standardized before group allocation. Lambing records later showed singleton, twin and triplet births, with one triplet‐bearing ewe in the nZnO group.

The control group received a basal total mixed ration (TMR), whereas the treatment group received the same ration supplemented with inorganic nano zinc oxide (Molchem NK007, 50 nm) at a dose of 25 mg/kg body weight (BW). This dose was selected to represent a moderate supplementation level within the biologically relevant range reported in small ruminant studies, while avoiding higher exposure during the transition period. Before the start of the experimental feeding protocol, a 15‐day adaptation period was applied. During the supplementation period, ewes assigned to the nZnO group were placed in individual feeding spaces, and the calculated daily dose of nZnO was top‐dressed onto the TMR immediately after feed delivery to facilitate individual consumption of the intended dose. The control group received the same basal TMR without nZnO supplementation.

The experimental period lasted 42 days. Rations for both groups were formulated according to NRC (2007) recommendations. All animals had ad libitum access to water and were fed twice daily. Repeated blood samples were collected from the same ewes at three physiological stages during the transition period: 21 days before lambing (prepartum), within 1–12 h after lambing and 21 days after lambing (postpartum). Baseline and post‐supplementation serum/plasma zinc concentrations were not determined; therefore, the zinc status of the animals could not be directly verified. This point was acknowledged as a limitation of the study. This study was approved by the Local Animal Experiments Ethics Committee. All procedures were conducted in accordance with relevant animal welfare regulations. In addition, written informed consent was obtained from the animal owner before the study was initiated.

### 2.2 Blood Sampling

Blood samples were collected from the jugular vein into 10 mL anticoagulant‐free tubes. The samples were centrifuged at 3500 rpm for 10 min, and the serum was separated and stored at −80°C until analysis.

### 2.3 Biochemical Analyses

Serum TAS was measured using a commercial kit (Rel Assay Diagnostics, Turkey) based on the automated colorimetric method described by Erel (2004). The assay measures the bleaching of the ABTS radical cation by antioxidants present in the sample. Measurements were performed on an automated chemistry analyser according to the manufacturer's instructions. The intra‐assay coefficient of variation reported by the manufacturer was below 3%. Results were expressed as µmol Trolox equivalent/L.

Serum TOS was measured using a commercial kit (Rel Assay Diagnostics, Turkey) according to the automated colorimetric method described by Erel (2005). In this assay, oxidants in the sample oxidize ferrous ions to ferric ions, and the ferric ions form a coloured complex with xylenol orange under acidic conditions. Calibration was performed using hydrogen peroxide standards. Results were expressed as µmol H_2_O_2_ equivalent/L.

OSI was calculated as the ratio of TOS to TAS × 100 and used as an integrated indicator of redox balance.

### 2.4 Metabolic Parameters

Additional serum biochemical variables were analysed to characterize energy, protein, lipid, mineral and liver‐related metabolism. Energy metabolism was assessed by NEFA and beta‐hydroxybutyrate (BHBA). Protein metabolism was evaluated by total protein, albumin and blood urea nitrogen (BUN). The lipid profile included total cholesterol and triglycerides. Liver‐related biochemical variables included alanine aminotransferase (ALT) and aspartate aminotransferase (AST), while mineral status was assessed by phosphorus and total calcium. All analytes were measured using species‐validated commercial biochemical kits (Randox Laboratories, United Kingdom) on an RX Daytona automated chemistry analyser (Randox, United Kingdom) according to the manufacturer's instructions.

### 2.5 Statistical Analysis

All statistical analyses were performed using IBM SPSS Statistics version 27.0 (IBM Corp., Armonk, NY, USA). Because repeated blood samples were obtained from the same ewes at three physiological stages during the transition period, the data were analysed using a linear mixed model for repeated measures. In this model, treatment group (control vs. nZnO), time (prepartum, lambing and postpartum) and the treatment × time interaction was included as fixed effects, whereas animal was treated as the repeated subject effect. When significant main effects or interaction effects were identified, pairwise comparisons were performed using the Bonferroni adjustment. Data are presented as mean ± standard deviation (SD), and a value of *p* < 0.05 was considered statistically significant. Because of the limited number of animals in each group, the study should be considered exploratory and primarily suitable for detecting large treatment‐related effects. Therefore, non‐significant results, particularly for oxidative stress indices, were interpreted cautiously.

### 2.6 Use of AI Tools

No artificial intelligence tools were used in the generation of study data, the conduct of analyses or the interpretation of results. They were used solely for language editing during the preparation of the manuscript. All scientific content, comments and the final text were reviewed and approved by the author.

## 3 Results

The oxidative stress findings are presented in Table 1. No significant effects of treatment, time or treatment × time interaction was detected for TOS, TAS or OSI (all *p* > 0.05). Although some numerical differences were observed between the control and nZnO groups at individual time points, these differences were not statistically supported by the mixed model and were therefore not interpreted as evidence of a significant treatment effect. The treatment effect for TAS approached but did not reach statistical significance (*p* = 0.094). The serum albumin, ALT, AST and BHBA results are shown in Table 2. Albumin concentrations remained within a relatively narrow range throughout the study, although a significant effect of time was detected (*p* = 0.006). Similarly, ALT and AST were significantly influenced by time (both *p* < 0.001), whereas neither the overall treatment effect nor the treatment × time interaction was significant. ALT values were higher in the prepartum period and lower at lambing, while AST values increased in the postpartum period in both groups. These findings indicate that temporal variation, rather than nZnO supplementation, accounted for the main changes in albumin and liver‐related enzyme activities. For BHBA, a significant overall treatment effect was observed (*p* = 0.009), whereas the effects of time and treatment × time interaction were not significant.

**TABLE 1:** Serum TAS, TOS and OSI values in Lacaune ewes during the transition period.

Groups	TOS (µmol/L)/Mean (±SD)	TAS (µmol/L)/Mean (±SD)	OSI
Control prepartum	7.94 ± 2.70	1139.08 ± 216.54	0.72 ± 0.26
Control parturition	7.64 ± 1.62	1083.59 ± 356.40	0.84 ± 0.58
Control postpartum	8.64 ± 2.66	1194.86 ± 302.87	0.74 ± 0.21
nZnO prepartum	4.70 ± 2.27	1254.96 ± 172.72	0.39 ± 0.20
nZnO parturition	6.72 ± 1.58	1267.96 ± 262.00	0.55 ± 0.17
nZnO postpartum	6.38 ± 1.51	1370.70 ± 245.93	0.49 ± 0.20
*p* (treatment)	0.852	0.094	0.745
*p* (time)	0.088	0.251	0.164
*p* (treatment × time)	0.110	0.858	0.866

*Note*: Values are expressed as mean ± SD. *p* values represent the effects of treatment, time and treatment × time interaction.

Abbreviations: OSI, oxidative stress index; TAS, total antioxidant status; TOS, total oxidant status.

**TABLE 2:** Serum albumin, ALT, AST and BHBA concentrations in Lacaune ewes during the transition period.

Groups	Albumin (g/dL)	ALT (IU/L)	AST (IU/L)	BHBA (mmol/L)
Control prepartum	2.96 ± 0.06	22.46 ± 3.35	114.07 ± 18.03	0.38 ± 0.06
Control parturition	2.98 ± 0.09	15.00 ± 6.40	99.43 ± 19.95	0.47 ± 0.14
Control postpartum	2.96 ± 0.05	16.57 ± 4.53	147.36 ± 28.48	0.48 ± 0.14
nZnO prepartum	2.98 ± 0.06	20.77 ± 4.47	99.71 ± 11.63	0.45 ± 0.05
nZnO parturition	3.02 ± 0.06	14.34 ± 2.48	97.00 ± 15.19	0.42 ± 0.08
nZnO postpartum	2.94 ± 0.06	15.40 ± 3.43	143.64 ± 18.35	0.45 ± 0.13
*p* (treatment)	0.471	0.837	0.117	0.009
*p* (time)	0.006	< 0.001	< 0.001	0.090
*p* (treatment × time)	0.331	0.884	0.318	0.081

*Note*: Values are expressed as mean ± SD. *p* values represent the effects of treatment, time and treatment × time interaction.

Abbreviations: ALT, alanine aminotransferase; AST, aspartate aminotransferase; BHBA, beta‐hydroxybutyrate.

**FIGURE 1:**
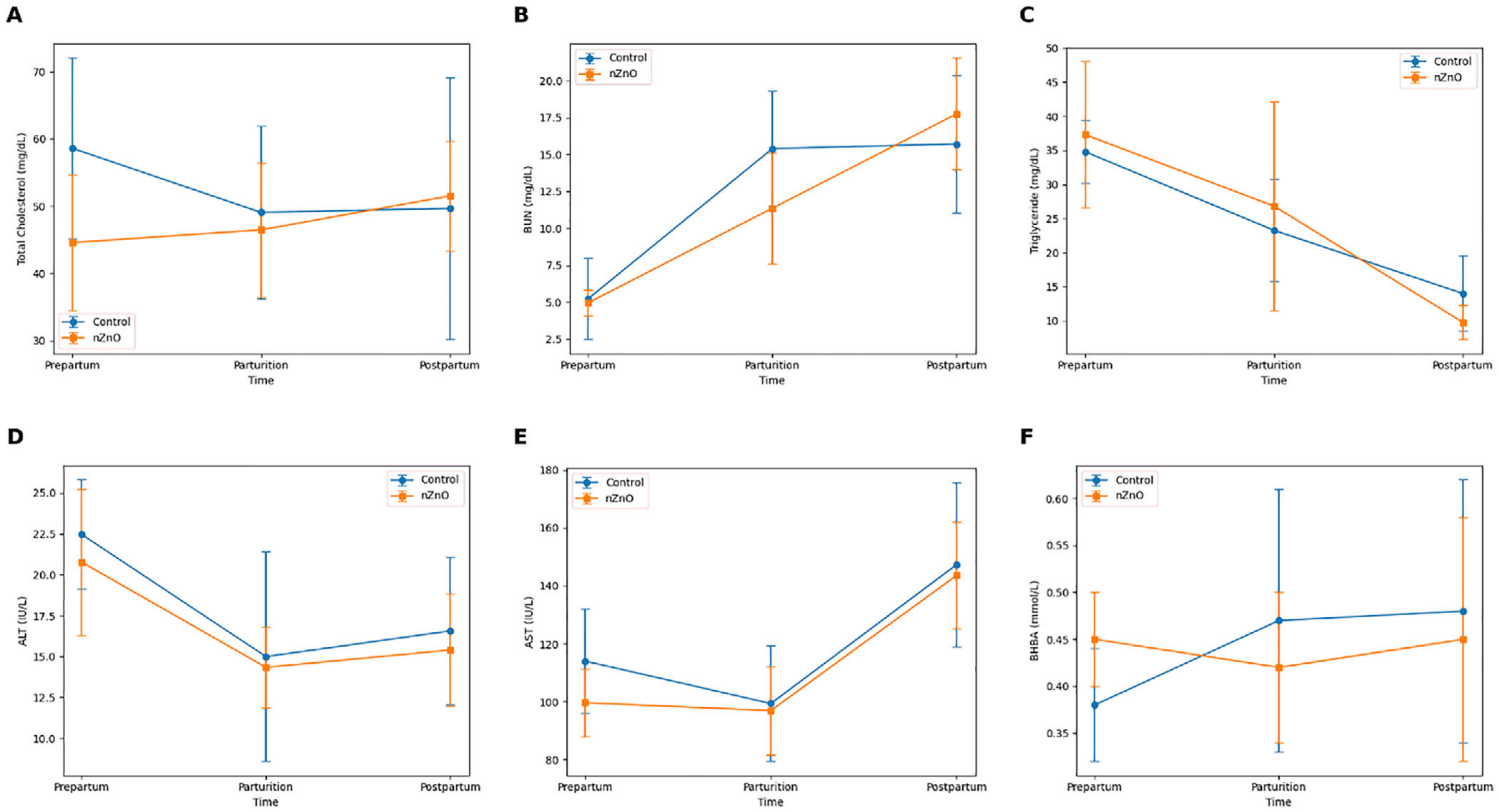
Temporal changes in selected metabolic and biochemical parameters in Lacaune ewes during the transition period. (A) Total cholesterol, (B) BUN, blood urea nitrogen, (C) triglyceride, (D) ALT, alanine aminotransferase (E) AST, aspartate aminotransferase and (F) BHBA beta hydroxybutyrate concentrations (mean ± SD) in control and nZnO (nano zinc oxide) groups across prepartum, parturition and postpartum periods. Significant effects of treatment, time or their interaction are reported in the corresponding tables.

As presented in Table 3, BUN concentrations were significantly affected by time (*p* < 0.001), with lower values in the prepartum period and higher values at lambing and postpartum. Phosphorus showed a significant overall treatment effect (*p* = 0.035), whereas no significant effect of time or treatment × time interaction was detected. NEFA concentrations were not significantly affected by treatment, time or their interaction. Therefore, the numerical differences observed for NEFA were not interpreted as evidence of altered lipid mobilization. The results for total calcium, total cholesterol, total protein and triglycerides are summarized in Table 4. Significant effects of time were found for total calcium, total protein and triglycerides (all *p* < 0.001), whereas no overall treatment effects or treatment × time interactions were identified for these variables. In contrast, total cholesterol showed a significant overall treatment effect (*p* = 0.035) as well as a significant treatment × time interaction (*p* = 0.033), indicating that the temporal pattern of total cholesterol differed between the two groups. Among the metabolic variables evaluated, BHBA, phosphorus and total cholesterol were the main parameters showing significant treatment‐related effects. The overall temporal trends and group differences for selected metabolic parameters are summarized in Figure 1.

**TABLE 3:** Serum BUN, phosphorus and NEFA concentrations in Lacaune ewes during the transition period.

Groups	BUN (mg/dL)	P (mg/dL)	NEFA (mmol/L)
Control prepartum	5.24 ± 2.76	5.13 ± 1.21	0.78 ± 0.31
Control parturition	15.39 ± 3.88	5.00 ± 0.89	0.81 ± 0.28
Control postpartum	15.70 ± 4.67	4.29 ± 1.41	1.16 ± 0.64
nZnO prepartum	4.96 ± 0.89	4.31 ± 1.11	0.60 ± 0.20
nZnO parturition	11.36 ± 3.75	3.95 ± 1.19	0.76 ± 0.23
nZnO postpartum	17.74 ± 3.78	3.73 ± 0.67	0.87 ± 0.44
*p* (treatment)	0.143	0.035	0.415
*p* (time)	< 0.001	0.353	0.061
*p* (treatment × time)	0.070	0.815	0.543

*Note*: Values are expressed as mean ± SD. *p* values represent the effects of treatment, time and treatment × time interaction.

Abbreviations: BUN, blood urea nitrogen; NEFA, non‐esterified fatty acids.

**TABLE 4:** Serum total calcium, total cholesterol, total protein and triglyceride concentrations in Lacaune ewes during the transition period.

Groups	T. Ca (mg/dL)	T. Cholesterol (mg/dL)	T. Protein (g/dL)	Triglyceride (mg/dL)
Control prepartum	9.97 ± 0.15	58.61 ± 13.43	6.09 ± 0.63	34.79 ± 4.65
Control parturition	10.04 ± 0.68	49.06 ± 12.84	5.37 ± 0.65	23.26 ± 7.48
Control postpartum	10.19 ± 0.21	49.66 ± 19.48	5.86 ± 0.31	14.01 ± 5.52
nZnO prepartum	10.00 ± 0.22	44.58 ± 10.10	6.26 ± 0.51	37.30 ± 10.75
nZnO parturition	10.34 ± 0.56	46.47 ± 10.01	5.00 ± 0.57	26.81 ± 15.33
nZnO postpartum	10.15 ± 0.18	51.51 ± 8.13	5.92 ± 0.61	9.78 ± 2.55
*p* (treatment)	0.457	0.035	0.187	0.206
*p* (time)	< 0.001	0.134	< 0.001	< 0.001
*p* (treatment × time)	0.375	0.033	0.122	0.337

*Note*: Values are expressed as mean ± SD. *p* values represent the effects of treatment, time and treatment × time interaction.

Abbreviations: T. Ca, total calcium; T. cholesterol, total cholesterol; T. protein, total protein.

## 4 Discussion

Zinc is an essential trace mineral that participates in a wide range of cellular and physiological functions. Adequate zinc supply is therefore necessary for the maintenance of normal metabolic, immune and reproductive activity in animals (Li et al. 2025; Sadegzadeh Sadat et al. 2021). Its importance becomes even more evident during periods of increased physiological demand. Experimental evidence has shown that zinc deficiency shortly before ovulation can impair embryonic development and increase the likelihood of pregnancy loss (Tian et al. 2014).

In the present study, nZnO supplementation did not significantly affect TOS, TAS or OSI during the transition period. Although numerically lower TOS and OSI values and higher TAS values were observed in some stages of the nZnO group, these differences were not statistically supported by the mixed model and should not be interpreted as evidence of a confirmed antioxidative effect. Similar improvements in antioxidant capacity have been reported in ruminants receiving nano forms of zinc (Abdelnour et al. 2021; Abdollahi et al. 2020; Alijani et al. 2020; Hosseini‐Vardanjani et al. 2020). Zinc contributes to the activity of several antioxidant enzyme systems, and its supplementation has been linked with improved antioxidant status in both dairy cattle and sheep (Chen et al. 2023; Hosseini‐Vardanjani et al. 2020; Khorrami et al. 2024; Klein et al. 2025; Li et al. 2025; Mir et al. 2020). The relatively large surface area and increased bioavailability of nano zinc oxide may further enhance these biological effects (Abdelnour et al. 2021; Swain et al. 2016). However, under the present experimental conditions, the tested nZnO dose did not produce a statistically measurable change in the global oxidative stress indices evaluated. This may be related to the limited sample size, the single‐dose design, the absence of direct zinc status assessment or the considerable physiological variation associated with the transition period. It is also possible that the dose or duration of supplementation was insufficient to overcome the metabolic variability of late pregnancy, lambing and early lactation. Therefore, the redox‐related findings should be interpreted as exploratory rather than confirmatory.

The liver‐related biochemical findings also support a cautious interpretation. Albumin, ALT and AST were significantly affected by time, whereas treatment and treatment × time interaction effects were not significant. Therefore, these changes appear to reflect temporal physiological variation during the transition period rather than a direct effect of nZnO supplementation. In particular, AST activity increased in both postpartum groups, suggesting that the metabolic demands of the early lactation period had a stronger effect than supplementation alone. Accordingly, the present findings should not be interpreted as evidence of hepatocellular protection by nZnO. Since only one supplementation level was tested, it is not possible to separate the contribution of zinc from the physiological burden associated with lambing and the onset of lactation. Previous studies in lambs and laboratory animals have shown that prolonged exposure or high doses of zinc oxide nanoparticles can impair liver integrity and increase serum transaminase activity (Najafzadeh et al. 2013; Pei et al. 2022; Tang et al. 2016). In contrast, practical supplementation levels used in sheep have not consistently been associated with adverse changes in liver related enzymes, indicating that dose, duration and physiological stage are likely to determine safety outcomes (Hosseini‐Vardanjani et al. 2020; Mohamed et al. 2017). Because breed‐, stage‐ and laboratory‐specific reference intervals were not directly evaluated in this study, liver enzyme changes were interpreted relatively across groups and sampling times rather than as definitive clinical evidence of hepatic dysfunction or protection.

Among the metabolic variables, BHBA, phosphorus and total cholesterol were the main parameters showing significant treatment‐related effects. BHBA showed a significant overall treatment effect, whereas time and treatment × time interaction were not significant. This finding suggests that nZnO supplementation may have been associated with differences in ketone‐body‐related energy metabolism across the study period. However, because NEFA was not significantly affected and zinc status was not directly measured, this result should be interpreted cautiously and should not be considered conclusive evidence of improved energy balance. Previous evidence indicates that zinc supplementation may influence glucose and energy metabolism in late pregnant ewes (Sadegzadeh Sadat et al. 2021), but further studies including direct zinc measurements, larger sample sizes and multiple supplementation levels are needed to clarify this relationship. In contrast to BHBA, NEFA concentrations were not significantly affected by treatment, time or their interaction. Therefore, the numerical NEFA pattern observed in the present study should not be interpreted as evidence of reduced lipid mobilization. Earlier studies in ruminants have shown that nano zinc supplementation may improve performance, rumen fermentation and nutrient utilization (Abdollahi et al. 2020; Alijani et al. 2020; Hosseini‐Vardanjani et al. 2020; Petrič et al. 2024). A similar relationship between oxidative stress biomarkers and metabolic indicators such as NEFA and BHBA has also been described in periparturient dairy goats (Meli et al. 2025). Nevertheless, the present NEFA data do not provide statistical support for a treatment‐related change in adipose tissue mobilisation.

The protein‐related variables also require conservative interpretation. Although total protein and BUN varied across physiological stages, these changes were not supported by significant treatment effects. Therefore, the present data do not allow a firm conclusion that nZnO improved nitrogen utilization or protein turnover. Comparable improvements in biochemical profile, antioxidant status and immune related responses have been reported in sheep or dairy cattle supplemented with zinc sources (Alijani et al. 2020; Klein et al. 2025; Mansour et al. 2023; Mohamed et al. 2017). Since zinc is involved in numerous enzymatic pathways related to tissue maintenance, protein metabolism and immune regulation, future studies may further investigate whether nZnO affects protein metabolism during late gestation and early lactation. However, the current findings should be viewed as preliminary and stage‐dependent rather than as evidence of a clear protein‐metabolic benefit (Mir et al. 2020; Yatoo et al. 2013).

The significant treatment effect on phosphorus may indicate that nZnO supplementation was associated with changes in mineral metabolism. However, because serum zinc concentrations, total dietary mineral intake and mineral interactions were not directly evaluated, the biological basis of this phosphorus response remains uncertain. Mineral absorption in ruminants may be influenced by interactions among dietary minerals and other dietary components within the digestive tract (Goff 2018). In addition, responses of serum phosphorus to nano zinc supplementation appear to be inconsistent across small‐ruminant studies; for example, nano zinc supplementation increased serum zinc but did not alter serum phosphorus in Malabari kids (Reddy et al. 2023). Therefore, the phosphorus finding in the present study should be considered a metabolic signal requiring further investigation rather than a basis for practical supplementation recommendations.

Comparisons among studies should be made with caution. Supplementation protocols vary considerably across experiments. Some studies express zinc oxide nanoparticle intake relative to BW, whereas others calculate inclusion on a dietary dry matter basis. In addition, differences in breed, physiological stage, feeding regime and basal mineral status may substantially influence the magnitude of the response (Abd El Rahim et al. 2023; Ibrahim et al. 2025; Mohamed et al. 2017). These methodological differences may partly explain the heterogeneity reported in the literature and support the need for dose optimisation studies in dairy type breeds such as Lacaune.

The lipid related findings of the present study were also stage dependent. Triglyceride concentrations were highest in the nZnO group during the prepartum period and lowest in the same group during the postpartum period. Stage related variation in serum lipid indices has previously been described in ewes across pregnancy, postpartum and lactation, suggesting that changes in triglyceride and cholesterol concentrations may reflect physiological adaptation as well as nutritional influences (Piccione et al. 2009). In the present study, total cholesterol showed both a significant treatment effect and a significant treatment × time interaction. This indicates that the temporal pattern of cholesterol differed between groups and that cholesterol was one of the few metabolic variables showing a statistically supported treatment‐related response. However, given the small sample size, single‐dose design and lack of zinc status assessment, this finding should be interpreted cautiously and confirmed in larger studies.

### 4.1 Study Limitation

A limitation of the present study is the relatively small number of animals included in each treatment group. Although repeated measurements were obtained from the same ewes at three physiological stages, the study should be considered exploratory and primarily suitable for detecting large treatment‐related effects. Consequently, smaller differences between groups may have remained undetected. This consideration is particularly relevant for the oxidative stress variables, for which no significant effects of treatment, time or treatment × time interaction was detected. Therefore, the lack of statistical significance for some variables should not necessarily be interpreted as definitive evidence of the absence of a biological response.

Another important limitation is that baseline and post‐supplementation serum/plasma zinc concentrations were not determined. Therefore, the initial zinc status of the animals and the biological uptake of nZnO supplementation could not be directly verified. In addition, only a single dose of nZnO was tested, and the study design did not allow dose‐response relationships or safety margins to be defined. Litter size was also not standardized before group allocation, which may have contributed to physiological variation during the transition period.

Furthermore, mechanistic markers related to antioxidant enzyme activity, mineral interactions and zinc‐dependent metabolic pathways were not evaluated. For this reason, the significant findings observed for selected metabolic variables, including BHBA, phosphorus and total cholesterol, should be interpreted as preliminary metabolic signals rather than conclusive evidence of a defined mechanism. Further studies involving larger numbers of animals, multiple supplementation levels, direct zinc status monitoring and broader mechanistic and safety assessments are warranted to clarify the magnitude, consistency and practical relevance of the observed responses.

## 5 Conclusion

At the tested dose, nZnO supplementation did not significantly alter oxidative stress indices, including TAS, TOS and OSI, in pregnant Lacaune ewes during the transition period. Significant treatment‐related effects were observed for selected metabolic variables, particularly BHBA, phosphorus and total cholesterol, suggesting possible metabolic responses rather than a confirmed improvement in oxidative balance. However, these findings should be interpreted cautiously because of the small sample size, single‐dose design, absence of direct zinc status assessment and the physiological variability associated with the transition period. Therefore, the present data do not support a firm recommendation for routine nZnO supplementation as a strategy to improve oxidative status in transition‐period Lacaune ewes. Further studies involving larger animal populations, multiple supplementation levels, direct zinc monitoring and broader safety and mechanistic assessments are required before practical nutritional or clinical recommendations can be made.

## Author Contributions

Conceptualization: G.B., C.O. Methodology: G.B., N.H.A. Investigation: G.B. Formal analysis: N.H.A. Writing – original draft: G.B., C.O. Writing – review and editing: G.B., N.H.A., C.O. All authors read and approved the final manuscript.

## Funding

The authors have nothing to report.

## Ethics Statement

The present study was approved by the Local Animal Experiment Ethics Committee of Aksaray University on 18 April 2023, under protocol number 2023/5‐19. The experiment was carried out on a commercial sheep farm situated in the central district of Aksaray Province, Türkiye. In addition, written consent was obtained from the animal owner before the study was conducted.

## Conflicts of Interest

The authors declare no conflicts of interest.

## Data Availability

Data are available from the corresponding author on reasonable request.
